# Recent Advances in Cellulose-Based Hydrogels for Tissue Engineering Applications

**DOI:** 10.3390/polym14163335

**Published:** 2022-08-16

**Authors:** Chao Chen, Yuewei Xi, Yunxuan Weng

**Affiliations:** 1 College of Chemistry and Materials Engineering, Beijing Technology and Business University, Beijing 100048, China; 2 Beijing Key Laboratory of Quality Evaluation Technology for Hygiene and Safety of Plastics, Beijing Technology and Business University, Beijing 100048, China

**Keywords:** cellulose, cellulose derivative, tissue engineering, cellulose-based hydrogel

## Abstract

In recent years, cellulose has attracted much attention because of its excellent properties, such as its hydrophilicity, mechanical properties, biodegradability, biocompatibility, low cost and low toxicity. In addition, cellulose and its derivatives contain abundant hydrophilic functional groups (such as hydroxyl, carboxyl and aldehyde groups), which are good raw materials for synthesizing biocompatible hydrogels. In this paper, the application prospects of cellulose and its derivatives-based hydrogels in biomedical tissue engineering are summarized and discussed through the analysis of recent research. Firstly, we discuss the structure and properties of cellulose, nano celluloses (NC) from different sources (including cellulose nanocrystals (CNC), cellulose nanofibrils (CNF) and bacterial nano celluloses (BNC)) and cellulose derivatives (including cellulose ethers and cellulose esters) obtained by different modification methods. Then, the properties and preparation methods of physical and chemical cellulose hydrogels are described, respectively. The application of cellulose-based hydrogels as a tissue engineering scaffold (skin, bone and cartilage) in the biomedical field is introduced. Finally, the challenges and prospects of cellulose-based hydrogels in tissue engineering are summarized.

## 1. Introduction

Defects in, or the loss of function of, tissues and organs are a few of the most common public health challenges, caused by many factors, such as trauma, disease, birth defects and aging, etc. [1,2]. It is necessary to treat the damaged area to facilitate the tissue regeneration process. Organ transplantation is the main technology for the treatment of various tissue and organ injuries. However, organ transplantation is limited by the lack of donors and the high cost of the process, and many people are on the transplant waiting list every year [3]. In addition, there are serious immune rejection reactions in organ transplantation. Tissue engineering, a new field of medical science, has emerged as an effective way to repair, improve or replace damaged tissues and organs. Tissue engineering was originally defined in 1988 as, “applying the principles and methods of engineering and life sciences to understand the structure–function relationship of normal and pathological mammalian tissues and to develop biological substitutes for repairing or regenerating tissue or organ function” [4]. Tissue engineering has a wide range of applications, such as the regeneration of skin, blood vessels, muscles and bones. Tissue engineering consists of three elements, namely, scaffolds, cells and growth factors [5]. Scaffolds play an integral role in tissue regeneration by providing structural support in three-dimensional (3D) space, attaching necessary nutrients and growth factors, and allowing the exchange of metabolites and gases to adapt and guide cell growth to specific tissues. In addition, this scaffold exhibits characteristics, such as biocompatibility, non-toxicity, biodegradability and mechanical strength. In the past few decades, researchers have explored and adjusted different combinations of different materials to make scaffolds that mimic natural tissues and organs [6,7].

**Figure 1:**
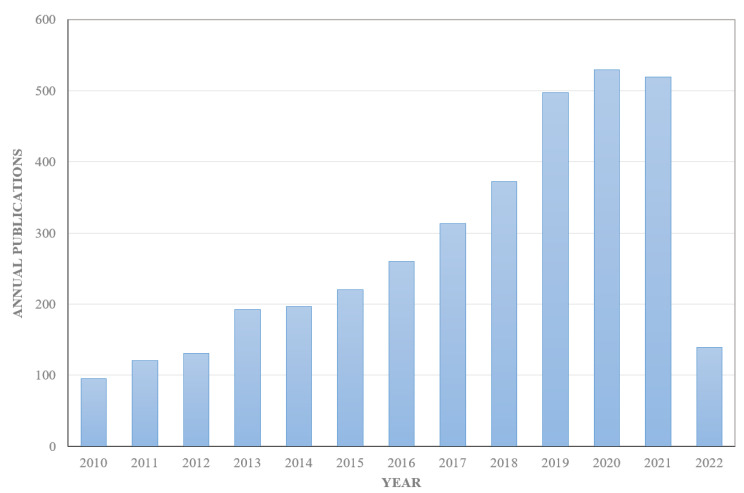
Annual publications from 2010 to 2022. Search Web of Science for the terms: “cellulose” and “tissue engineering”.

In tissue engineering, hydrogel has attracted more and more attention for its unique and excellent properties. Hydrogel is a 3D polymer material that continues to absorb and expand when it comes into contact with water. The compatibility of hydrogel comes from its high water content. Hydrogels have a similar structure to natural extracellular matrices, allowing nutrients, growth factors and cellular waste to diffuse through elastic networks, making them ideal materials for tissue engineering [8]. However, the application of hydrogels in the biomedical field has limitations. When synthesizing hydrogels, the removal of toxic crosslinkers is time-consuming and leaves residues [9]. In addition, the use of most non-biodegradable and non-recyclable synthetic raw materials poses a threat to environmental sustainability [10]. Nowadays, being green and renewable are important concepts in every research study, therefore, there is a need to use a natural resource—natural hydrogel materials that can be easily modified into biocompatible hydrogel products. Natural hydrogel materials include polysaccharide-based materials, such as cellulose, chitosan and alginate; and protein-based materials, such as silk fibroin, collagen, elastin and gelatin. Among these materials, cellulose is reported to be the most widely distributed natural source material on earth, with a biomass production of about 1.5 × 10^12^ tons per year [11,12]. Cellulose is a fibrous, tough and water-insoluble substance that plays an important role in maintaining the structure of plant cell walls and is widely found in plants, animals (such as tunicates), algae, fungi and minerals in nature [13,14]. Therefore, the source material is abundant and easy to produce. Cellulose is considered an ideal tissue engineering scaffold material due to its high mechanical strength, reproducibility, good biocompatibility, biodegradability and non-toxicity [15]. At the same time, this is evidenced by the increasing number of papers published on the application of cellulose materials in tissue engineering (Figure 1). In addition, due to the abundance of hydroxyl groups in cellulose molecules, it can be used to prepare hydrogels with different structures and properties as a platform for advanced tissue engineering and regenerative medicine. 

In this review, we summarize the application of cellulose-based hydrogels in various tissues (skin, bone and cartilage) from the perspective of tissue engineering. Firstly, the basic structure and properties of cellulose and its derivatives are analyzed and the information on cellulose-based hydrogels are studied. Then we briefly review the latest research progress on cellulose-based hydrogels in tissue engineering. Finally, cellulose composites as a new generation of tissue engineering materials are summarized and prospected.

## 2. Basic Properties of Cellulose and Its Derivatives

### 2.1. Chemical Structure and Properties of Cellulose

Cellulose is the most abundant biodegradable material in nature, mainly distributed in natural plants in the form of microfibers, accounting for more than 50% of the carbon content in the plant kingdom [16]. It is also found in some animals (e.g., tunicates), and in small amounts in algae, fungi, bacteria and minerals. Cellulose was first discovered and prepared by Payen in 1838, and since then all aspects of its physical and chemical properties have been thoroughly studied.

**Figure 2:**
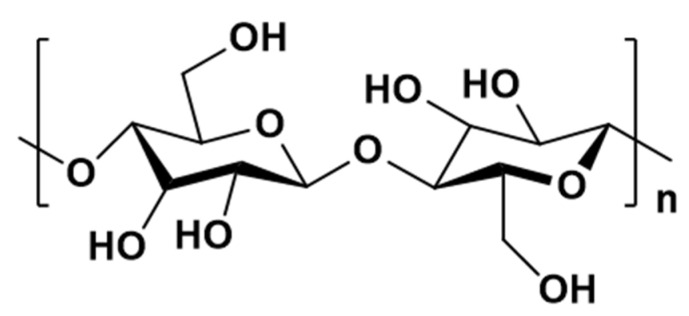
The molecular structure of cellulose.

The structure of cellulose is hierarchical, resulting in different physical properties through different structural isotopes and assemblies of basic microfibrils [17]. The molecular formula of cellulose is (C_6_H_10_O_5_)_n_, where n is determined by the degree of polymerization and is generally more than 2000–15,000 [18], depending on the structure and source of cellulose. The linear and rigid cellulose molecular chain consists of D-glucopyranose ring units, each linked by beta-1, 4-glycosidic bonds (see Figure 2), in which a carbon atom from a gluconic anhydride ring is cross-linked by forming a covalent bond with a carbon atom from a neighboring gluconic anhydride ring via an oxygen atom. Its chemical composition contains 44.44% carbon, 6.17% hydrogen and 49.39% oxygen [19,20]. Each cellulose chain has two distinct end groups: one with a chemically reduced functional group (hemiacetal unit) and the other with a lateral hydroxyl group, which is the nominal non-reduced end [21]. Due to the different accumulation and aggregation of cellulose chains, the structures of different cellulose-producing organisms are also different, resulting in the complex structure of cellulose [22]. Cellulose has three hydroxyl groups in each glucose residue. This hydrophilic structure enables it to have strong water absorption and good reactivity. In terms of physical structure, cellulose is a fibrous, multi-capillary polymer, with porous and large surface area characteristics, so cellulose has a certain capacity for adsorption.

From the chemical structure of cellulose, there are a large number of highly polar hydroxyl groups on the glucopyranosyl ring, which are very beneficial to the formation of intramolecular and intermolecular hydrogen bonds. Intramolecular hydrogen bonds form the linear structure of cellulose chains, while intermolecular hydrogen bonds form the fiber structure and crystallization region, which together ensure a high mechanical strength and flexibility of cellulose [23]. In addition, thanks to the synergy of intermolecular hydrogen bonds, hydrophobic action and van der Waals forces, the parallel accumulation of cellulose chains is facilitated, which further assembles into fibers. Individual cellulose chains assemble to form crystalline regions scattered in amorphous regions [24]. In the crystalline region, the molecular chains of cellulose are regularly arranged, the intermolecular force is larger, the hydrogen bonding energy between the molecular chains is high, the orientation of the molecular chains is good, and the density is larger, so the crystallization region of cellulose enables cellulose to have higher strength. In the amorphous region, the arrangement of macromolecules is not neat and is relatively relaxed, the number of hydrogen bond bindings between molecules is small and the density is low.

**Figure 3:**
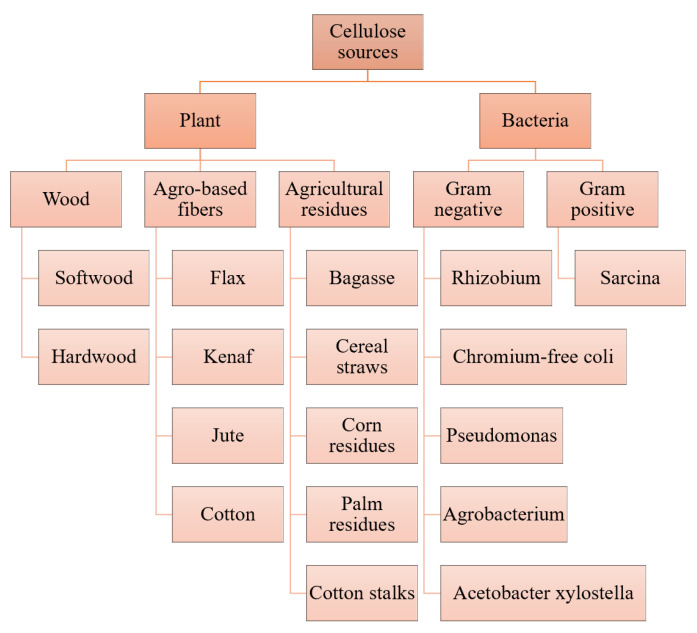
Fiber classification by source: plant and bacteria.

Cellulose, a semi-crystalline material, has a crystallinity that depends on its source, the extraction method and its pretreatment [25]. According to its source, cellulose is mainly derived from plant cellulose (PC) and bacterial cellulose (BC). Common sources of cellulose are shown in Figure 3. The PC can be divided into wood, agro-based fibers and agricultural residues. wood cellulose is usually extracted from softwood (such as pine, cedar and spruce) and hardwood (such as oak and beech); agro-based fibers are usually extracted from flax, kenaf, jute and cotton. Agricultural residues are usually extracted from bagasse, cereal straws, corn residues, palm residues and cotton stalks. The cellulose content in plants is generally between 30% and 75%, and the cellulose content in wood is generally between 40% and 50% [25]. However, cellulose is often combined with hemicellulose and lignin, and the complex junction structure between lignin and hemicellulose forms a matrix around the cellulose molecule that is so tough that it is difficult to break [26]. Therefore, during the isolation of cellulose, these compounds must be removed or at least eliminated.

Although obtaining pure cellulose from plants requires different chemical treatment methods, BC is easily produced in its pure form [11] because BC is absent from hemicellulose and lignin, which makes cellulose easy to extract and purify. The synthesis process of BC consists of: (1) the glucose chain integrating the fibrils; (2) the secretion of fibrils at the synthetic site on the cell wall; (3) the aggregation of the resulting fibrils into microfibers; and (4) the further organization of microfibers into bundles [27]. As shown in Figure 3, common BC sources are mainly produced extracellularly by Gram-negative bacteria such as *Acetobacter xylantobacter KJ1*, *Agrobacterium tumefaciens*, alkali-producing bacteria, *pseudomonas*, *rhizobia*, *sucrose bacillus*, chromium-free bacteria and *rhizobia*, as well as produced by Gram-positive bacteria, such as *Octadiae*. As shown in Table 1, the significant differences between BC and PC are in their purity, crystallinity, water retention, mechanical properties and porosity [17]. PC has a moderate water retention capacity of 60% and has a moderate level of tensile strength and crystallinity. However, BC’s high chemical purity (BC does not contain hemicellulose and lignin, with a cellulose content of about 100%), crystallinity, porosity, water absorption (up to 400 times its dry weight), water retention (up to 98%), as well as its excellent 3D microfiber network structure, large specific surface area and extraordinary mechanical strength (especially under wet conditions) make BC a promising biomedical material [28,29]. Hydrogels are composed of a 3D network of hydrophilic polymers containing large amounts of water and can be considered promising hydrogels in their natural state, due to the high moisture content and the stable mechanical properties of BC [30,31]. More importantly, BC is considered to be a non-cytotoxic and highly biocompatible material, attracting interest in the biomedical field [32].

### 2.2. Structure and Properties of Cellulose Derivatives

**Figure 4:**
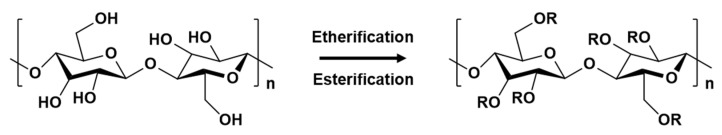
Cellulose is etherified or esterified to form cellulose derivatives.

The chemical structure of cellulose shows that each glucose molecule has three free hydroxyl groups, which have a good ability to form intermolecular and intramolecular hydrogen bonds [33]. Cellulose has a crystalline and rigid structure due to the tight connections between the entangled cellulose chains, resulting in cellulose being almost insoluble in water and in most organic solvents, as well as a lack of thermoplasticity [34]. Researchers often use different solvent systems to dissolve cellulose, among which ionic liquids (ILS) are considered to be environmentally friendly solvents, including NaOH/urea, NaOH/thiourea, etc. [35]. However, there are limitations to its use, such as its high energy consumption, high cost and inherent difficulties in solvent recovery. To improve the solubility of cellulose and expand its application, controlled chemical or physical surface modification is necessary to improve the properties of cellulose [36]. Cellulose can be modified into different cellulose derivatives by destroying the ordered crystallization region due to the large number of hydrogen bonds between hydroxyl groups. As shown in Figure 4, cellulose derivatives mainly include etherification (cellulose ether) and esterification (cellulose ester). Derivatives may differ in basic properties, such as chemical structure, hygroscopicity, water interaction, surface activity and solubility.

**Figure 5:**
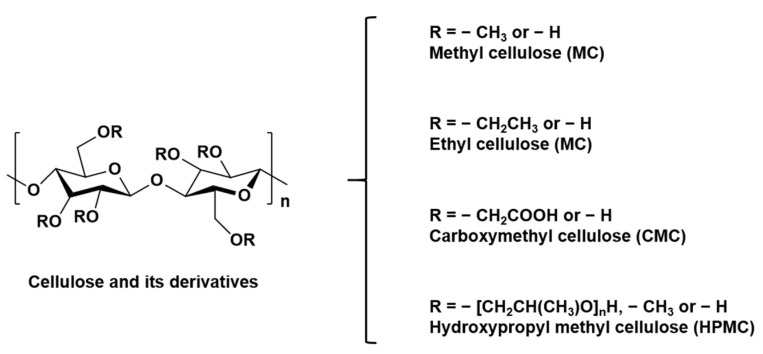
The structural formula of methyl cellulose (MC), ethyl cellulose (EC), carboxymethyl cellulose (CMC) and hydroxypropyl methyl cellulose (HPMC).

Cellulose ethers: cellulose hydroxyl groups can be partially or fully etherified by various reagents, such as epoxides and alkyl halides. Cellulose ethers are mostly water-soluble cellulose derivatives, and their water solubility depends on the chemical structure of the substituent, and the degree and mode of substitution. This class of water-soluble cellulose derivatives possesses several favorable properties, such as solubility, solution viscosity, surface activity and good stability against oxidative, thermal and biological degradation [37,38]. Furthermore, the hydrophilicity, mechanical toughness, pH stability and thermogelling ability of cellulose ethers are believed to stimulate wound healing [39]. The most widely used cellulose ethers in the biomedical field are: carboxymethyl cellulose (CMC), methyl cellulose (MC), ethyl cellulose (EC) and hydroxypropyl methyl cellulose (HPMC) [40], and their structural formulas are shown in Figure 5.

CMC is a commercialized cellulose ether and one of the lowest-priced polymers, featuring hydrophilicity, water-solubility, nontoxicity, biocompatibility and biodegradability. CMC is formed by the reaction of cellulose with monochloroacetic acid, in which the hydroxyl groups on C_2_, C_3_ and C_6_ of each glucose residue are replaced by carboxymethyl groups. The optimal degree of substitution for CMC in biomedicine is usually between 0.6 and 1.25. In addition, the distribution of carboxymethyl substituents in the polymer chain also affects the properties of CMC. Sodium carboxymethyl cellulose (NaCMC), a sodium salt of CMC, is an anionic polymer with a good solubility in water [41]. NaCMC is often used as an important component of wound dressings because it can absorb a large amount of exudate and avoid skin tissue dehydration and tissue necrosis [42].

EC and MC are cellulose derivatives with similar chemical structures, and they are widely used commercial cellulose ethers. EC is formed by the reaction of alkali cellulose with ethyl chloride and is usually distinguished by its viscosity, relative molecular mass and degree of substitution. EC is soluble in most organic solvents, such as ethanol, methanol and chloroform, if the degree of substitution is between 2.2 and 2.6. If that degree of substitution is high than 2.8, EC becomes highly insoluble. EC has a good strength at room temperature, but its strength decreases immediately with increasing temperatures [43]. In addition, EC is a biodegradable substance with the advantages of hydrophobicity, low toxicity and thermoplasticity [44], and its excellent film-forming ability and appropriate mechanical properties make it an excellent material to use in different industries [45]. MC is formed by the etherification reaction of cellulose molecules with methyl chloride or dimethyl sulfate in an alkaline solution, with a low crystallinity [46]. MC is soluble in a variety of organic solvents, depending on the degree of substitution. In addition, MC undergoes reversible sol-gel transition thermosensitive behavior in an aqueous solution with temperature change [47]. MC has excellent biocompatibility and biodegradability and is widely used in biomedical applications.

HPMC is a white, fibrous or granular powder made by substituting the hydroxyl groups of cellulose molecules with methyl and hydroxypropyl groups. Due to the thickening, gelling and swelling properties of HPMC and the temperature response of HPMC-based hydrogels [48], this cellulose derivative can be widely used in controlled release directions [49]. Because of its low toxicity, biocompatibility, biodegradability, excellent stability, good surface activity and mechanical properties, it has been widely used in the biomedical field.

Cellulose ester: cellulose can be esterified with acetic acid, nitric acid and phosphoric acid to produce water-insoluble esters [36]. Cellulose esters have good solubility in common solvents and will melt before decomposition. Generally speaking, cellulose esters can be divided into two categories: organic and inorganic, of which organic cellulose esters are more widely used in the medical field [50]. The first commonly used organic cellulose ester is cellulose acetate (CA). Synthesis methods of CA include ring-opening esterification and transesterification under heterogeneous or homogeneous conditions, as well as esterification using imine chloride or N, N-carbonyl diimidazole [51]. In addition, current CA preparation techniques do not require further chemical or mechanical treatment of the remaining cellulose, which is beneficial for biomedical applications, such as tissue engineering and drug delivery systems. Some of the remarkable properties of CA, including good mechanical properties, hydrophobicity, biodegradability, biocompatibility, non-toxicity and low cost are characteristics associated with its natural origin. Cellulose nitrate, also known as nitrocellulose or celluloid, is the main ingredient in smokeless powder and has been widely used since the 20th century. Nitrocellulose is produced by the reaction of cellulose and nitric acid, replacing the cellulose hydroxyl group with a nitro group, and its nature and application depend on the degree of nitrification [25]. Cellulose sulfate is a cellulose ester obtained through heterogeneous, homogeneous or quasi-homogeneous sulfate processes, and is a cellulose derivative with a relatively simple chain structure and unique biological properties [52]. Due to the existence of sulfate groups, compared with pure cellulose, cellulose sulfate has excellent properties, such as water solubility, antibacterial and anticoagulant properties at high concentrations [27]. In addition to its simple preparation and low cost, cellulose sulfate has good biocompatibility and biodegradability, which makes it a potential tissue engineering application material [53].

### 2.3. Structure and Properties of Nanocellulose

Due to the close arrangement of cellulose molecular chains, the intermolecular and intermolecular hydrogen bonding forces lead to the existence of a huge binding energy of cellulose molecules, which makes cellulose difficult to dissolve in general solvents. Although cellulose can be dissolved in a special solvent to form a homogeneous cellulose solution, the hydrogen bonds between cellulose molecules are destroyed during the dissolution process, and the crystal structure of cellulose is also transformed from highly regular cellulose I to cellulose II. The strength of cellulose-II-type crystal is relatively low, so the strength and modulus of regenerated cellulose are lower than those of natural fibers. In addition, most cellulose solvents are toxic. Therefore, in addition to traditional cellulose and its derivatives, there is also innovative cellulose and its derivatives, namely nanocellulose (NC) [54]. NC is a kind of cellulose in nanometer form, which decomposes natural cellulose from large units (μm) to small units (nm) by breaking the chain between intramolecular and intermolecular hydrogen bonds [55]. It can not only be well-dispersed in water to form NC hydrogel but also retains the crystal structure and hydrogen-bond interaction of natural cellulose I, with high strength, high modulus and other characteristics [56]. NC broadly refers to cellulose having at least one spatial size in the range of 1 to 100 nm. Compared with cellulose, NC has a larger specific surface area, higher length-diameter ratio, better strength and greater modification flexibility. The high length-diameter ratio of NC leads to a strong physical entanglement, and meanwhile, the hydrogen-bond interaction also promotes cellulose to form a three-dimensional network structure. In addition, NC has super-hydrophilic properties and a large specific surface area and can absorb a large amount of water to form NC hydrogel with good mechanical properties. The gelation process is a reversible process; when a certain external force is applied or the external conditions are changed, the structure of the NC hydrogel is destroyed, and when the external mechanical force is removed, the structure of the NC hydrogel is reconstructed. Higher concentrations are required for the formation of hydrogels with NC, with surface charges prepared by oxidation or other pre-treatments, since the repulsion between the surface charges of the NC hinders their entanglement.

**Figure 6:**
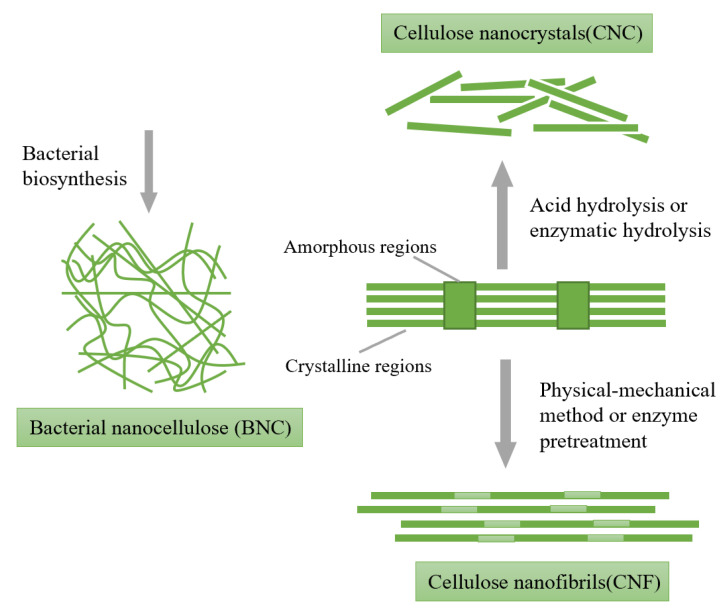
Different types of nanocelluloses: nanocrystalline cellulose, nanofibril cellulose and bacterial nanocellulose. Data reproduced from ref. [61].

As shown in Figure 6, NC is mainly divided into three types: rigid rod-like cellulose nanocrystals (CNC), long and soft cellulose nanofibrils (CNF) and high purity and crystallization of bacterial nano cellulose (BNC) [57]. Although these types of NCs have relatively similar chemical compositions, NCs differ in crystallinity, size and morphological characteristics due to different sources and production techniques [58]. CNC and CNF are mainly obtained by degrading plant fibers [59]. The Canadian Standards Association (CSA) defines CNF as a fiber object composed of at least one predominant fiber, which is composed of a crystalline region and an amorphous region with a length to width ratio usually greater than 50. CNC is a rigid rod-like particle with a diameter of 10–30 nm and a length of hundreds of nanometers, which is mostly crystalline [60]. CNFs, also called microfibrillated cellulose, nanofibrils, nanofibrillated cellulose (NFC) or microfibrillated cellulose, are mainly prepared by the physical-mechanical method and physical-chemical combination method [61]. The physical-mechanical method mainly degrades cellulose through physical action, mainly using two methods: the first method is the high-pressure homogenization method, in which various forces such as high pressure, shear force, velocity and turbulence are generated by a rapid change of pressure to shear fibrils and make them stratified; the second is the physical grinding method, which mainly grinds the fibers into cellulose in the nano-size range through a sufficiently long grinding time and energy. In addition, there are several different stratification devices, such as microfluidic machines, steam blasters, ultrasonic and high-speed mixers, etc. [62]. CNF prepared by the physical-mechanical method increases the specific surface area after the fiber is highly refined, exposing a large number of hydroxyl groups on the surface, thus showing good hygroscopicity and adhesion [63]. Although the physical-mechanical method is relatively simple, it usually leads to problems, such as fiber breakage, excessive degradation or uneven degradation, so the CNF produced has the disadvantages of low polymerization degree, low crystallinity and low aspect ratio. At the same time, the physical-mechanical method has a very high energy consumption and the use of long fibers will lead to mechanical congestion. Therefore, the physical-mechanical method has certain limitations in practical application, which also derives a physical-chemical binding method for preparing CNF by pretreating the fiber raw material with chemical reagents or cellulase and then combining it with the above physical methods. Pretreatment can effectively reduce the energy consumption of physical CNF preparation and enhance the colloid stability of the final CNF [4]. At present, the main methods of pretreatment are cellulase treatment [64], 2,2,6,6-tetramethylpiperidine nitrogen oxide radical (TEMPO) oxidation and carboxymethylation [65]. Compared with CNC, CNF has a significant tendency to form entangled networks due to its high aspect ratio and semi-crystalline structure, which is beneficial to the formation of hydrogels with higher mechanical stability. In addition, CNF can be added into the hydrogel as a filler to improve the flexibility of the hydrogel.

CNC, otherwise called cellulose nanowhiskers or cellulose crystallites, are mainly prepared by chemical methods [61]. The chemical method includes acid hydrolysis and enzymatic hydrolysis. The chemical method can reduce energy consumption and enhance fibrillation compared with physical methods. The acid hydrolysis method refers to the hydrolysis of natural cellulose under acidic conditions, and the β-(1–4)-D-glucoside bond of cellulose macromolecule is broken, destroying the amorphous region and low crystallization region in the fiber, to extract cellulose nanocrystalline [6,66]. The most commonly used is sulfuric acid [43], in addition to hydrolysis by hydrochloric acid [67], phosphoric acid [68] and nitric acid [69], etc. The choice of acid directly affects the thermal stability, size and surface charge of CNC. Although the acid hydrolysis method is simple and time-saving, it comes with the problems of corrosion of equipment, pollution of the environment and low CNC yield. Due to the limitations of acid hydrolysis, the researchers found that CNC could also be obtained by enzymatic hydrolysis. Enzymes can catalyze the hydrolysis of cellulose and enhance its fibrosis due to the directional and catalytic functions of enzymes. However, this method takes a long time, the production cost of the enzyme is high and the raw material needs to be pretreated, so it has not been used in large-scale production. In general, due to the small aspect ratio and the rigid structure of CNC, it lacks the ability to intertwine to form mechanically stable hydrogels. Mechanically stable CNC hydrogels are prepared by altering the surface chemistry or incorporating it into the polymer hydrogel network. 

Microorganisms can produce BC directly [70]. The most commonly used microorganism is acetic acid bacteria of the genus Gluconobacter (same name Komagataeibacter). Bacterial nano cellulose (BNC) is a stable network of water-containing nanofibers composed of 1% cellulose and 99% water, with a diameter between 20 and 100 nm [60]. BNC production methods range mainly from the stirred culture method to the static culture method using batch or fed-batch culture and continuous culture. In most cases, BNC is produced by the culture of acetic acid bacteria in an aqueous culture medium under static conditions. Different bacterial strains, media composition, temperature, pH and other parameters have a significant impact on productivity, so this method can be tailored to the desired application of BNC materials. As BNC is considered to be a highly biocompatible material, we can see an increasing number of publications focusing on the medical applications of BNC [15]. It is worth mentioning that wound dressings made from BNC are currently on the market and have even entered clinical studies.

Manipulating cellulose molecules and their supramolecular aggregates in the nanometer size range, designing and assembling new nanomaterials with excellent functions, have become frontier approaches in the field of cellulose science [71]. The large number of hydroxyl groups on the surface of NC provide NC with the nature of its hydrophilicity, which allows NC to easily agglomerate itself, resulting in a low utilization rate in nanocomposites. Therefore, to improve the poor dispersibility of NC or to obtain a nanocellulose derivative having the desired properties, researchers have generally chemically modified NC in various manners, such as the acetylation of NC, silanization, graft copolymerization modification, surface-active agent modification and surface modification, in order to introduce specific groups, effectively prevent particle agglomeration, increase its stability and dispersibility and meet the needs of various practical applications.

## 3. Cellulose-Based Hydrogels

Hydrogels have unique and excellent properties, and their use as biomaterials is closely related to their properties. Hydrogels are special dispersion systems with a spatial network structure in which the dispersed phase is water and the solid phase is a solid three-dimensional (3D) network [72]. Hydrogels can absorb and retain large amounts of water in an aqueous environment by swelling under the action of surface tension and capillary forces [73,74]. The high water content and physicochemical/mechanical properties that are similar to natural extracellular matrices make hydrogels ideal candidates as biocompatible materials for use in in vivo applications [8]. In addition, the hydrogels are reasonably deformable and suitable for the type of surface to be applied; they have strong tissue adhesion, which can promote wound healing, coupled with their soft mechanical properties: elasticity and swelling capacity, hydrogels have emerged as excellent tissue engineering scaffolds for biomedical applications [75,76]. Hydrogels based on cellulose and its derivatives have biodegradability, high hydrophilicity and good mechanical strength, which are widely used in tissue engineering, such as skin, muscle, bone, cartilage and blood vessels. However, cellulose hydrogels differ from other hydrogels in that the polymers they form are insoluble in water, whereas most other hydrogels are soluble [77]. On the one hand, the insolubility of cellulose can be solved by modification; on the other hand, the physical entanglement and electrostatic action of the NC fiber network can be used to stabilize the structure, and chemical crosslinking can further enhance the performance of the structure.

Crosslinking is the formation of a bond or connection between two polymer chains. Crosslinking is the most critical step in the preparation of hydrogels, which turns the polymer solution into a “gel” (solid) by restricting the movement of the polymer chains. Thus, the 3D structure of the hydrogel is maintained, and the mechanical properties of the hydrogel are improved. Three key parameters characterize the overall structure of the hydrogel: (i) a measure of the relative molecular mass of the polymer chain between two crosslinks, i.e., the degree of crosslinking (CD), independent of the type of bonding that occurs, CD being a determinant of the porosity of the hydrogel, which reflects the change in pore size (ε), diffusion coefficient and correlation length [10]; (ii) the volume fraction of the polymer in the swollen state, which is a measure of the amount of water retained by the hydrogel; (iii) The mesh size is defined by the structural parameter “ξ”, which represents the linear length between two adjacent crosslinks [38,78]. The design and synthesis of hydrogels can be carried out by adjusting the structure of hydrogels, such as the degree of crosslinking, mechanical strength, chemical reaction and response of hydrogels to stimuli [35]. There are two kinds of cross-linking methods for synthesizing hydrogels, namely physical and chemical methods, and the generated hydrogel is, respectively, a physical hydrogel and a chemical hydrogel. 

**Figure 7:**
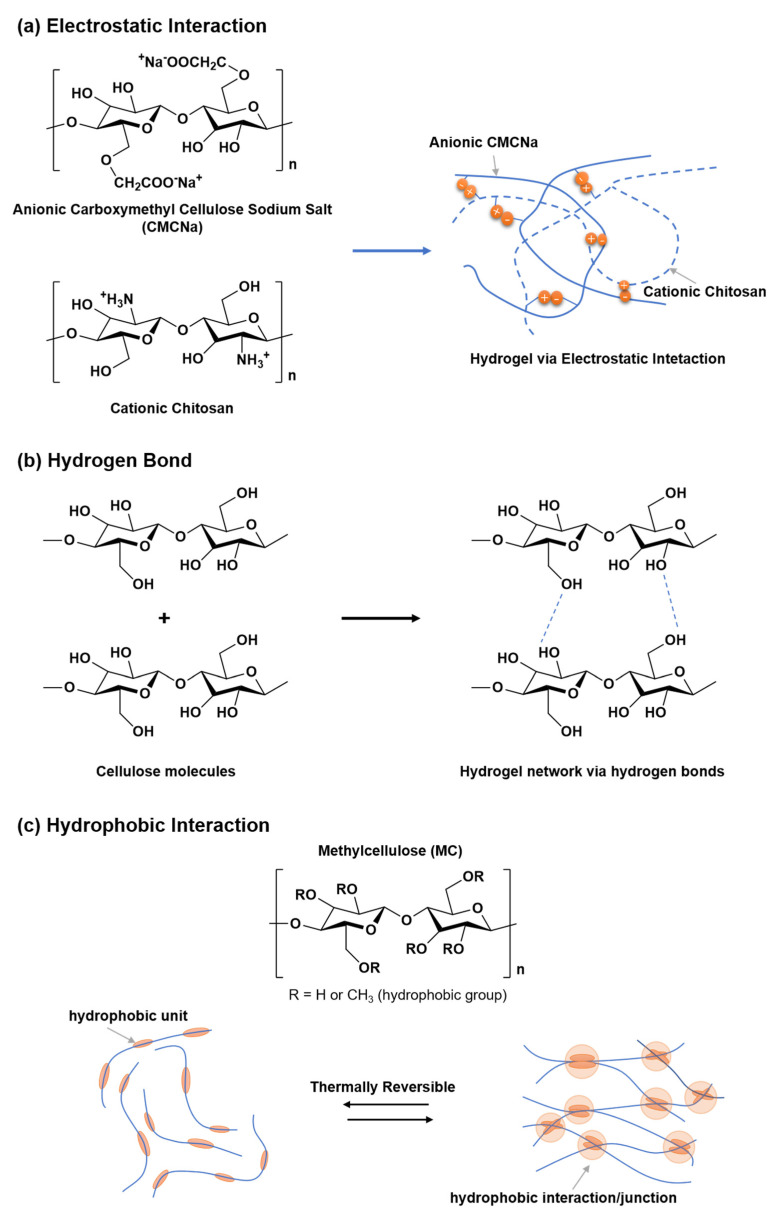
Crosslinking mechanism of physical hydrogels via (**a**) electrostatic interactions, (**b**) hydrogen bond interactions, and (**c**) hydrophobic interactions. Data reproduced from ref. [83].

Physical or reversible hydrogels are defined as hydrophilic polymer networks held together by the physical entanglement of polymer chains or other non-covalent interactions [79]. Common physical hydrogel cross-linking mechanisms are shown in Figure 7. These physical forces include macromolecular chain entanglement, hydrogen bonding, electrostatic interactions, van der Waals interactions, hydrophobic forces and ionic forces, and these reversible interactions are easily disrupted [80]. Therefore, the main advantage of physical cross-linking hydrogels is that they are easy to process and avoid the use of toxic cross-linking agents. However, one of the main disadvantages of physical hydrogels is that they are generally weaker in mechanical properties compared to chemical hydrogels. Zhang et al. [81] developed a dual physically crosslinked CMC-Fe^3+^/polyacrylamide (CMC-Fe^3+^/PAAm) dual-network hydrogel by a simple two-step process. In this hydrogel, Fe^3+^ cross-linked CMC forms the first network, while hydrophobic association PAAm forms the second network to maintain the integrity of the hydrogel. This gel has good mechanical properties (tensile strength is 1.82 MPa; toughness is 6.52 MJ/m^3^). In addition, due to the reversibility of the dual physical cross-linking interaction, the resulting hydrogels exhibit excellent self-healing and fatigue resistance properties. Appel et al. [82] prepared polymer nanohydrogels by electrostatic interaction using CMC, hyaluronic acid (HA) and a positively charged surfactant (cetyltrimethylammonium bromide), and the resulting self-assembled hydrogels were shear-thinning and self-healing.

Chemical or irreversible hydrogels are obtained by covalent cross-linking between cellulosic polymer chains using suitable chemical cross-linking agents. Covalent bonds between polymer chains may be formed using polyfunctional groups in the polymerization step, or by the reaction of side groups or side chains in a post-polymerization process. The physicochemical properties of chemically crosslinked hydrogels depend on the degree of crosslinking, hydrogel composition and stimuli responsiveness (sensitivity to pH, temperature, light and magnetic fields, etc.) [84], and it has viscoelastic stability and the advantage of a high mechanical strength. Chemical cross-linking agents are essential elements of chemical hydrogels, which react with active functional groups of cellulose and/or its derivatives to form cross-linking networks. Common chemical crosslinking agents include succinic anhydride (SA), citric acid (CA), epichlorohydrin (ECH), glutaraldehyde, ethylene glycol diglycidyl ether (EGDE) and divinyl sulfone (DVS). Alam et al. [85] reacted cellulose with sodium monochloroacetate (MCA) to obtain carboxymethyl cellulose (CMC) and cross-linked epichlorohydrin (ECH), and the water retention value (WRV) of the newly prepared hydrogel reached 725 g d-water/g gel, which is significantly higher than other commercial superabsorbent cellulose-based materials. However, toxic and carcinogenic by-products are produced when cross-linking with ECH, and researchers are committed to finding alternatives to toxic crosslinking agents for green applications. Zheng et al. [86] acidified CMC paste in a non-toxic CA solution and obtained self-healing CMC hydrogels. The healing efficiency of hydrogels with a compressive strength of 2.5 Mpa was 80%.

## 4. Application of Cellulose-Based Hydrogels in Tissue Engineering

The goal of tissue engineering is to construct artificial tissues/organs to effectively replace damaged tissues/organs in vivo and restore their functions [87,88]. Tissue engineering scaffolds play an important role in regeneration by mimicking the extracellular matrix (ECM) in natural tissues and providing a suitable 3D space for the growth of new tissues, thus promoting cell growth, proliferation and differentiation [89]. Therefore, scaffold materials must have reasonable biocompatibility, mechanical properties, swelling behavior and porosity. Due to its huge swelling capacity, adhesion, elasticity and 3D network structure, hydrogels can be used as scaffolds simulating ECM to encapsulate and deliver cells and promote cell organization and morphogenesis [90]. Cellulose and its derivatives have been widely used in biomedical applications due to their excellent biodegradability, biocompatibility, non-toxicity and functionality. Compared with other materials, the addition of cellulose and its derivatives from natural sources can greatly improve the mechanical properties of polymer matrices without sacrificing the biocompatibility and biodegradability of materials. As shown in Table 2, the cellulose-based hydrogel can greatly improve the mechanical properties of skin, bone and cartilage tissue engineering. The following will be detailed in these three parts.

### 4.1. Skin

The skin is the largest organ in the human body, with an area of 1.2 m^2^~2 m^2^, and its main function is to act as an external barrier to protect internal organs from the external environment (mechanical damage, radiation, chemical substances, bacteria and viruses, etc.) [97,98]. The skin is also a sensory organ, containing a large class of sensory neurons that transmit information about the environment to the brain [99]. In addition, the skin plays an important role in homeostasis, removing toxins, maintaining regular water levels, preventing electrolyte loss and controlling body temperature and blood pressure [39]. Skin wounds are a common occurrence in daily life and heal effectively in one to two weeks. Many systemic diseases cause skin damage, such as pathogens, pollution, smoking, malnutrition, obesity, diabetes, pressure sores, inflammation, bleeding and immunosuppression. In most cases, these factors lead to slow wound healing and easy recurrence, leaving severe scarring and causing serious annoyance to the patient. Skin tissue diseases are a worldwide public health problem. According to statistics, chronic non-healing wounds affect approximately 8.2 million Medicare beneficiaries, and Medicare costs for all wounds are projected to increase from $2.81 billion to $96.8 billion [100,101]. The global advanced wound-care market is projected to reach $18.7 billion by 2027, growing at a CAGR of 6.6% in 2020–2027. In addition, chronic wounds are more common in the elderly population, which the U.S. Government estimates will exceed 77 million by 2060, indicating that chronic wounds will continue to be an increasingly persistent problem in this population. In particular, chronic non-healing wounds are particularly vulnerable to infection with coronavirus disease (COVID-19) [101]. Therefore, reasonable wound treatment plays a vital role in repairing damaged tissue and improving the quality of life of patients with damaged wounds. At present, the clinical treatment mode for skin injury is skin transplantation, because the area of skin tissue available for transplantation in the human body is very limited, and this method is limited by aspects of hygiene and safety. As a result, conventional repair methods are very limited in the treatment of large skin lesions. The development of tissue engineering technology provides a new technical scheme for skin injury repair. Skin tissue engineering uses biological scaffolds that can be degraded and absorbed by the human body to carry cells. With the assistance of growth factors, while the functional cells in the complex continue to proliferate and differentiate, the scaffold gradually degrades and finally forms the same tissue as the original tissue. Among the different tissue engineering scaffolds, hydrogels are of particular interest because their structure is similar to the extracellular matrix, which can provide a moist environment and a porous structure. In addition, hydrogel has the ability of hydration healing, and has appropriate oxygen permeability while absorbing wound exudates, preventing bacterial infection, improving epithelialization and providing an environment for tissue regeneration [102]. Cellulose and its derivatives have excellent mechanical properties and a high water absorbency, and contain a large number of hydroxyl groups, which are conducive to the formation of composite hydrogels with other polymers or small molecules, and are good scaffolds materials for tissue engineering.

**Figure 8:**
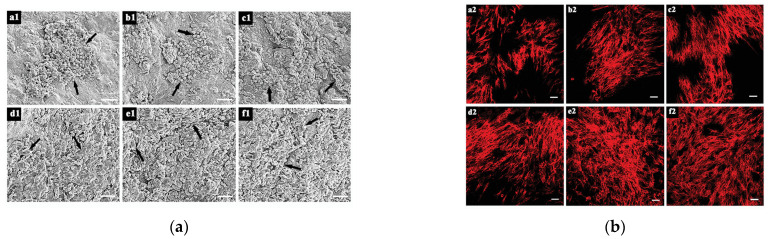
(**a**) SEM images of NIH3T3 cells after culturing on the rBC-based hydrogels for 3 days. The black arrow indicates NIH3T3 cells on different hydrogels. (**b**) LSCM images of NIH3T3 cells after culturing on rBC-based hydrogels for 7 days. In (a1–f1) and (a2–f2), each of the six images represents the six different groups, rBC hydrogel and rBC/MXene hydrogels with 0.1, 0.2, 0.5, 1 and 2 wt% of MXene, respectively. Scale bars are all 25 µm. Data reproduced from ref. [104].

**Figure 9:**
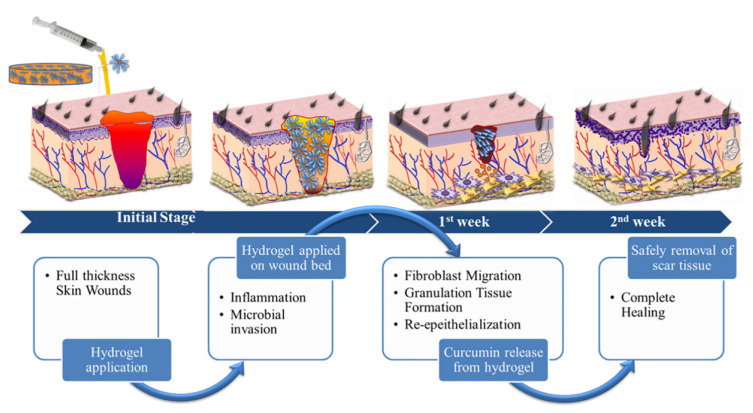
Wound healing mechanism at the same stage after hydrogel application. Data reproduced from ref. [107].

Excessive contraction of the wound can lead to scarring and fibrosis, which can affect healing. Nuutila et al. [103] compared the wound contraction of two different porcine full-thickness wounds with and without CNF or Purilon^®^ hydrogel (Coloplast, Humlebaek, Denmark) 14 days after surgery. The results showed that CNF hydrogel inhibited 70% of the puncture biopsy wound contraction, while Purilon^®^ hydrogel was ineffective. These results indicated that CNF hydrogel could be used as a new material to control excessive wound contraction. The skin is an electrically excitable tissue with a conductance between 2.6 and 1 × 10^−4^ mS cm^−1^ depending on the composition of the skin. To promote the electrical conductivity of a BC-based hydrogel, Mao et al. [104] prepared biodegradable and electroactive BC/MXene hydrogel containing MXene by the chemical and physical double crosslinking method based on the unique electrical conductivity and good biocompatibility of MXene(Ti_3_C_2_Tx), which was used to electrically regulate the cell behavior of skin wound healing in vitro. As shown in Figure 8, the adhesion and spread of NIH3T3 cells incubated on BC-based hydrogel for 3 days increased with the increase in MXene content. In a rat model of the full-thickness skin defect, the hydrogel showed a better therapeutic effect than the commercial Tegaderm membrane. Patele et al. [105] developed injectable and adhesive hydrogels for wound healing by incorporating spherical NC (s-NC) into a matrix of carboxymethyl chitosan (CMCs). The composite hydrogel scaffold was cultured with human dermal fibroblasts (HDF), human keratinocytes (HaCaT) and human umbilical vein endothelial cells (HUVEC) separately and co-cultured, respectively. The results showed that the hydrogel scaffold had good biocompatibility and had the potential to promote skin cell regeneration. In the scaffolds with s-NC, its antibacterial properties and wound healing effect were better than those of the control group. Liu et al. [106] prepared CNF by the TEMPO oxidation method, introduced polydopamine (PDA) into the CNF network, and prepared PDA/NFC hydrogel by physical calcium ion crosslinking with the calcium ion as the crosslinking agent. The hydrogel had a good pH/NIR response capability and could control drug release on demand at a lower pH or under near-infrared irradiation, with a maximum drug release ratio of 77%. In addition, the composite hydrogel can be successfully applied to skin wound dressing experiments and is a good tissue engineering material. CNC has a large specific surface area, which increases the connection point of the hydrogel and can be used as a nano reinforcement filler to improve the strength of the hydrogel. Huang et al. [91] produced CNC from wood cellulose by sulfuric acid hydrolysis, and then prepared dialdehyde-modified CNC (DACNC) by oxidation with periodic acid. A novel nanocomposite hydrogel was constructed by combining flexible carboxymethyl chitosan (CMC) chains with rigid DACNC, which was cross-linked by dynamic Schiff base bonds between the amine of CMC and the aldehyde of DACNC. The gel has a high self-healing efficiency (~5 min), high injectivity, good mechanical strength and an equilibrium swelling ratio of 350%. In addition, amino acids can alter the equilibrium of the Schiff base, resulting in the decomposition of the hydrogel, which can therefore be dissolved in the amino acid solution as needed, allowing painless removal when changing the wound dressing. In vivo experiments showed that the injectable self-healing hydrogel was effective in treating deep second-degree burn wounds, and only 0.6% of the wounds were not closed after healing for 2 weeks, and no scar was formed. Zhang et al. [92] proposed to use sodium alginate (SA)/NaCMC blend hydrogel as a bio-ink for artificial skin, where NaCMC could improve the mechanical strength of hydrogel scaffold and provide cell adhesion points. By studying different components, it was found that the mixed hydrogel with SA:NaCMC = 4:1 could achieve a repair effect that was similar to autologous skin transplantation. Cellulose provides a structurally stable 3D environment for cell growth and improved wound healing. Furthermore, TEMPO-oxidized cellulose nanofiber (TOCN) facilitates dispersion in aqueous solutions compared with unoxidized and bacterial cellulose. Shefa et al. [107] prepared a physical hydrogen bond cross-linked TOCN-polyvinyl alcohol (PVA)-curcumin (Cur) hydrogel by the freeze-thaw method, which could release Cur with antibacterial, anti-inflammatory and antioxidant functions to promote wound healing. It can be seen from Figure 9 that the wound healing mechanism of hydrogel occurs in different stages. After 2 weeks of treatment with hydrogel, obvious new epidermis and granulation tissue were formed in the defect area, and collagen fibers were gathered near the defect area, indicating that the TOCN-PVA-Cur hydrogel can effectively promote wound healing.

### 4.2. Bone

**Figure 10:**
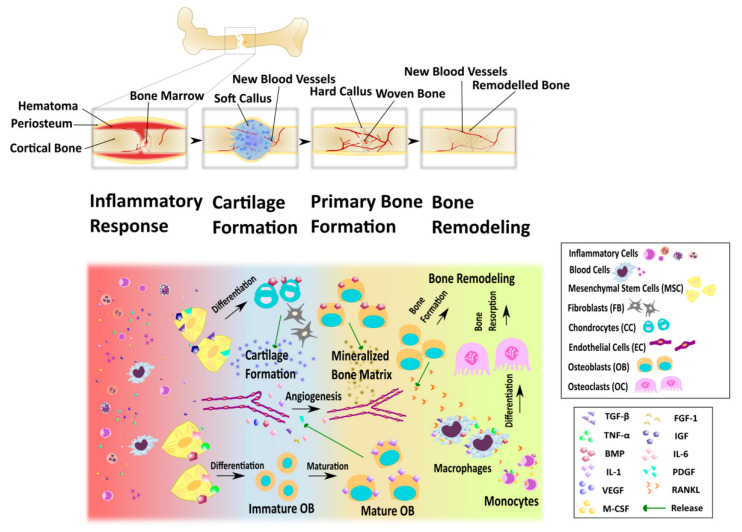
Schematic diagram of the stages of fracture healing. The upper part: the changes of bone tissue in the process of repair; the second part: some cellular aspects of bone repair and the factors involved in the bone repair process. Data reproduced from ref. [113].

Bones are complex organs composed of a specific-cell-type surrounded by an extracellular matrix (ECM) in which its bioactive molecules are integrated or produced by the cells [108]. The bone matrix usually accounts for more than 90% of the volume of bone tissue, and the rest is made up of cells and blood vessels. The bone ECM is composed of organic components (22 wt%, including collagen types I, III and IV and fibrin), inorganic crystalline mineral components (69 wt%, mainly calcium phosphate and hydroxyapatite) and water (9 wt%) [109,110]. The framework of cross-linked collagen fibers provides flexibility to the bone, while the inorganic components are located between the collagen fibers. This precise hierarchical structure gives the bone excellent toughness and strength [111]. Phosphate and calcium ions are stored in the bone and can be released into the blood when needed, thus bones have the function of producing blood and storing important minerals [112]. Another important function of bones is to support and ensure movement for the body, and to stabilize and protect internal organs. In humans, natural fracture repair is a complex process involving immune system activation, cell migration, differentiation and apoptosis, and bone tissue can undergo natural regeneration over time. The bone tissue repair process can be divided into four overlapping phases: inflammatory response, cartilage formation, primary bone formation and bone remodeling (Figure 10) [113]. Most bone injuries, such as fractures, defects and local necrosis, can be treated with conventional methods. For example, fractures can be treated by reduction, internal fixation (intramedullary nails), external fixation (splints and plaster bandages) and functional training [114]. However, bone growth is a dynamic process and bone defects exceeding a critical size threshold (usually >2 cm, depending on the anatomical site) will not heal naturally [115,116]. Severe bone injuries, such as severe traumatic fractures, degenerative diseases, congenital defects or osteosarcoma can cause large bone defects, and if functional recovery and complete healing are to be achieved, reconstruction with bone graft substitutes is required to promote the bone-healing process [117]. Many treatments, such as autogenous bone grafting and allograft bone grafting have been applied to the treatment of bone defects. Unfortunately, autogenous bone grafting has been associated with additional complications at the donor site, limited availability of autogenous bone and a scarcity of materials [118]. Side effects of bone allograft transplantation include immune rejection and the transmission of infectious pathogens [119]. Therefore, tissue engineering has attracted extensive attention in the field of bone tissue regeneration, and tissue engineering scaffolds integrating stem cells and bioactive molecules are becoming a feasible alternative [120]. The ideal scaffold for bone tissue engineering has three main properties: enhanced osteoconduction, osteoinduction and osteointegration [61]. Osteoconduction is the growth of the phalanx either on the surface of the implant or inward into pores, channels, or canals; osteoinduction describes the ability to attract pluripotent cells to the healing site and stimulate their development into osteogenic cells; osseointegration is a critical process in establishing stability between the phalanges and the implant [112,121]. Traditional scaffolds are mainly made of metal, calcium phosphate ceramic or glass, and have osteoconductive rather than osteoinductive properties [114]. In addition, they have the disadvantages of being prone to the inflammatory cascade, having a lack of biological recognition and stress shielding. Hydrogels are 3D network structures of natural or synthetic polymers that are considered to be very useful tissue engineering matrices due to their porosity. These cells can grow and proliferate, carry growth factors, and diffuse nutrients and drugs [122]. Interestingly, bone tissue engineering requires materials with different structures and properties to ensure the effectiveness of the designed solution, depending on the site of the injury and the patient’s physical condition. For example, bone tissue engineering programs in the elderly need to take into account age-related decline in bone regeneration potential, while bone tissue engineering materials used in pediatric patients require dynamic structural properties to adapt to continuous bone growth in patients [108,123]. Cellulose materials have biocompatibility, controllable degradation rate and excellent flexibility, which can meet the needs of bone tissue engineering scaffold materials and can be designed reasonably according to specific requirements.

**Figure 11:**
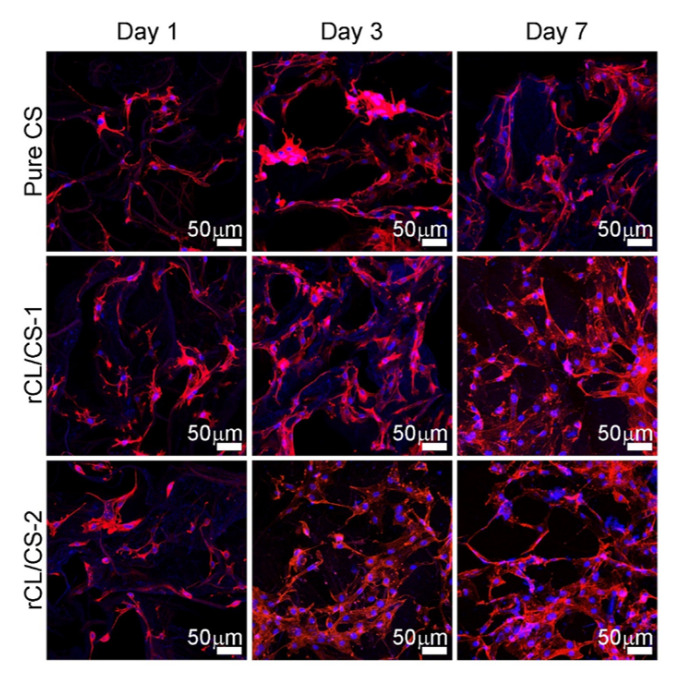
Confocal laser scanning microscopy (CLSM) microscopic images of MC3T3-E1 pre-osteoblast cells cultured on pure CS and rCL/CS hydrogel scaffolds. In the CLSM experiment, the density of MC3T3-E1 cells was maintained up to 3 × 10^4^ cells/well. The CLSM imaging was performed on days 1, 3 and 7. In CLSM, the nucleus and cytoskeleton of the cell were stained with DAPI (blue) and rhodamine (red), respectively. Data reproduced from ref. [93].

**Figure 12:**
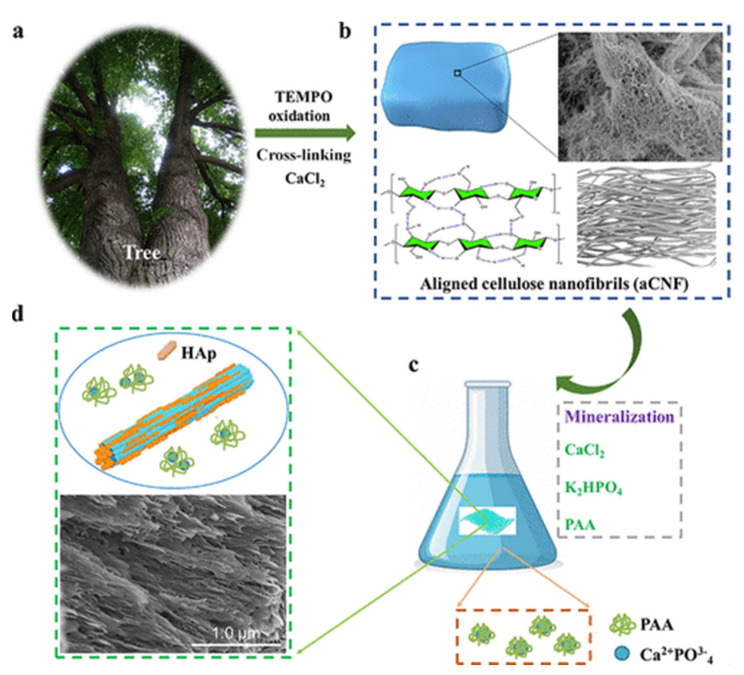
Schematic diagram of m-aCNF composite: (**a**) a tree; (**b**) aCNF and its structure; (**c**) Metallogenic processes; (**d**) The obtained m-aCNF material and its scanning electron microscope photographs show the oriented mineralized structure. Data reproduced from ref. [126].

The aqueous solution of MC has the properties of a thermosensitive gel, but the gel temperature of pure MC solution in vivo is too high (40–60 °C) to be used as an injectable hydrogel. Deng et al. [124] added nano-hydroxyapatite (nHA) into MC hydrogel by a physical blending method, and the gelation temperature of hybrid gel reached a more appropriate level (34 °C). ARS staining showed that the presence of nHA promoted osteogenic differentiation, and the repair efficiency of bone marrow mesenchymal stem cells loaded with the nHA hybrid MC hydrogel was improved in the skull defect repair experiment. Natural hydrogel scaffolds often have insufficient mechanical strength, and NC can be used to enhance the effect. Maharjan et al. [93] prepared regenerated cellulose (rCL) nanofibers by deacetylation of electrospun CA nanofibers and mixed them into a pure chitosan (CS) hydrogel to prepare rCL/CS composite hydrogel scaffolds. Compared with the pure CS, the compressive strength of the rCL/CS composite hydrogel was remarkably improved (the highest compressive strength could reach 30.19 kPa), and the activity, the attachment and the proliferation capability of the pre-osteoblast cell MC3T3-E1 were enhanced (Figure 11), and the osteogenic differentiation capability was improved. Therefore, rCL/CS composite hydrogel is expected to be a bone tissue engineering scaffold. To overcome the low mechanical properties of cellulose scaffolds and to improve the bioactivity of bone tissue regeneration, the mineralization of cellulose hydrogels with hydroxyapatite and other calcium phosphates has been actively investigated [125]. As shown in Figure 12, Qi et al. [126] prepared highly oriented CNF (aCNF) with TEMPO oxidation and moderate ultrasonic treatment. It was soaked in biomimetic mineralized solution (4.5 mM CaCl_2_, 4.2 mM K_2_HPO_4_, 50 mg/L 450 kDa polyacrylic acid (PAA)) for 28 days to obtain a fully mineralized aCNF hydrogel (m-aCNF). The entire m-aCNF fibers had a continuous mineral network, and the minerals were tightly packed and penetrated the aCNF. The m-aCNF nanohybrid materials have excellent mechanical properties, which are comparable to those of natural hard tissues, including human dentin and mouse cortical bone, under hydrated conditions. It is a promising material for bone tissue engineering. Traditional synthetic hydrogels not only have low mechanical strength and hardness but also have isotropic network structures. Inspired by that layered anisotropic structure of bone and wood, Wang et al. [127] delignified natural pine to obtain white wood (WW) with an aligned fibrous skeleton and impregnated sodium alginate (SA) hydrogel into the WW followed by in situ mineralization with a hydroxyapatite (HAp) nanocrystal to produce highly anisotropic, super strong, hard hydrogel composites. The well-aligned CNF leads to the highly anisotropic structure and mechanical properties of the composites. The hydrogen-bonding interactions between SA and WW, as well as the nano-reinforcement of the inorganic HAp phase, result in hydrogels with significant tensile strength (67.8 Mpa) and longitudinal elastic modulus (670 Mpa). In vitro, the composite material can effectively promote osteogenic differentiation; in vivo, it can induce bone formation and promote osseointegration, and become a promising candidate for bone repair. The limited mechanical properties of fibrous hydrogels have hindered their application in hard tissue regeneration. Basu et al. [94] prepared scaffolds by adding BC synthesized from *Xyloacetate bacilli CCM3611T* (*Acetobacter xylanate*) as the substrate into polyethylene pyrrolidone (PVP) and then adding calcium phosphate (CAp) synthesized from hydroxyapatite (HA) and β -tricalcium phosphate (β-TCP) on the above basis. The compressive strength of the obtained CAp/BC-PVP hydrogel scaffold was between 0.21~0.31 Mpa, which was equivalent to that of the human cancellous bone. In addition, significant cell activity was observed in cell viability studies in the human osteosarcoma Saos-2 cell line, indicating the ability of the hydrogel to promote cell growth and cell proliferation. In another study by Basu et al. [128], inorganic calcium fillers β-tricalcium phosphate (β-TCP), calcium carbonate (CaCO_3_) and hydroxyapatite (HA) were added to a BC hydrogel to improve the osteoconductive and osteoinductive properties of biomaterials, and it was found that fibroblasts (LEP-3) could effectively adhere and grow on the surface of BC-PVP-β-TCP/HA hydrogel scaffold. Therefore, the scaffold has good bone conduction performance.

### 4.3. Cartilage

**Figure 13:**
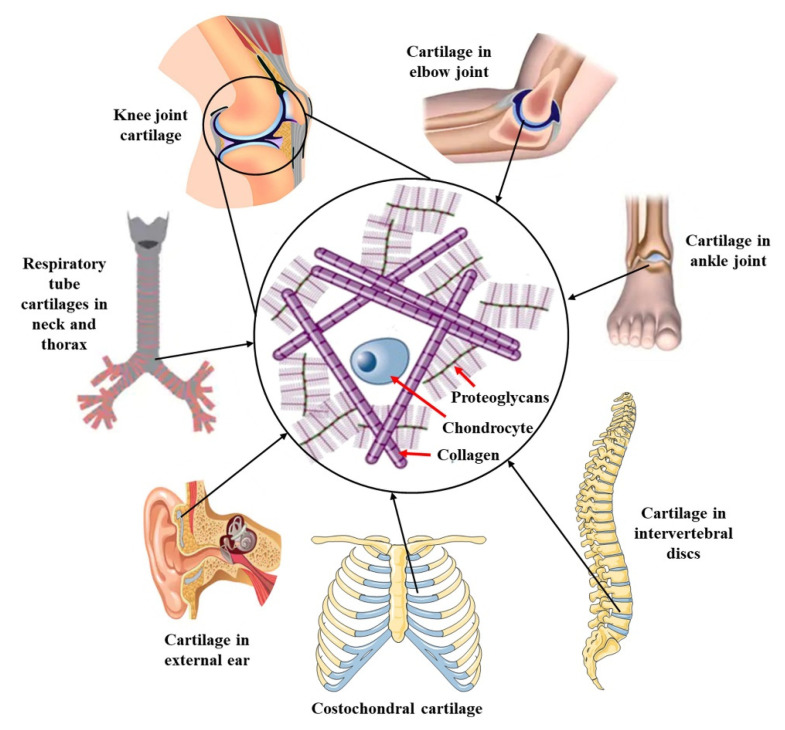
Diagram of cartilage tissue and its structure in different parts of the human body. Collagen (type II), proteoglycans and chondrocytes are the major components of cartilage tissue. Data reproduced from ref. [129].

Cartilage is a special kind of connective tissue. Hyaline cartilage is usually found in joints and has a thickness of about 1 mm to 3 mm, which varies from joint to joint [129]. As shown in Figure 13, cartilage tissue is widely present in various parts of the human body. Cartilage is responsible for transferring and relieving stress from one bone to another, and due to the high elasticity of bone, can cause reversible deformation during joint movement [130]. Therefore, cartilage has the functions of shock absorption, force transmission, friction reduction and joint stability [131]. Unlike most other tissues in the body, cartilage contains only one special type of cell, the chondrocyte. Chondrocytes are distributed in the cartilage ECM. Cartilage ECM is mainly composed of water (60–80% of total weight), collagen (60% dry weight) and proteoglycan (30% dry weight). Other materials remain, including lipids, non-collagenous proteins and other glycoproteins. The most important component of ECM is water, so the movement of water molecules in the network stabilizes cartilage tissue under stress [132]. Type II collagen is the most abundant isoform in articular cartilage, accounting for more than 80% of all collagens, and is considered to be a marker of chondrocyte differentiation along with proteoglycans. Proteoglycan resists tough and sustained shear and compressive load, maintaining tissue elasticity and durability; collagen is the major factor in the tensile strength and shape of tissues. Due to the absence of any lymphoid connections, nerves and blood vessels in the cartilage tissue, the cell migration ability is limited, resulting in a low endogenous healing ability, and therefore the self-repair ability of damaged cartilage tissue is limited [133]. When the damaged area is large (>4 mm), it does not heal on its own [134]. Once cartilage tissue is damaged, adjacent cartilage is more susceptible to wear. In addition, the injury can cause inflammation throughout the joint, which in turn can cause tissue degradation and damage. Thus, damage to articular cartilage tissue begins with a sports injury, accidental injury, or inflammation, and often progresses to osteoarthritis (OA) and rheumatoid arthritis (RA). Currently, 250 million people are affected by osteoarthritis due to increases in life expectancy, body mass index and joint damage [135]. The incidence of this degenerative disease increases with age and is a major health problem [136]. Common treatment approaches include direct chondrocyte replacement (including allograft cartilage transplantation and mosaicplasty), cell culture-based therapies (including matrix-induced autologous chondrocyte implantation and autologous chondrocyte implantation) and bone marrow stimulation techniques (including drilling, microfracture and abrasion) [129]. The main limitations of these methods are a mismatch of performance in the repaired area, morbidity in the donor site and lack of integration of the transplanted tissue [137]. In this context, cartilage tissue engineering has been achieved by using two main sources of chondrocytes: chondrocytes and mesenchymal stem cells, to repair lesions and damage to cartilage and to restore its normal function. The regeneration of cartilage essentially requires the use of appropriate scaffolds to restore its biological and mechanical properties. As a scaffold material for cartilage tissue engineering, the hydrogel has the advantages of high viscoelasticity, high water content and an ECM microenvironment simulating natural cartilage, and is expected to become a candidate material for cartilage regeneration [138]. Cellulose is a suitable scaffold material for tissue engineering because of its hydrophilicity, biocompatibility, non-toxicity and excellent binding ability with other materials.

**Figure 14:**
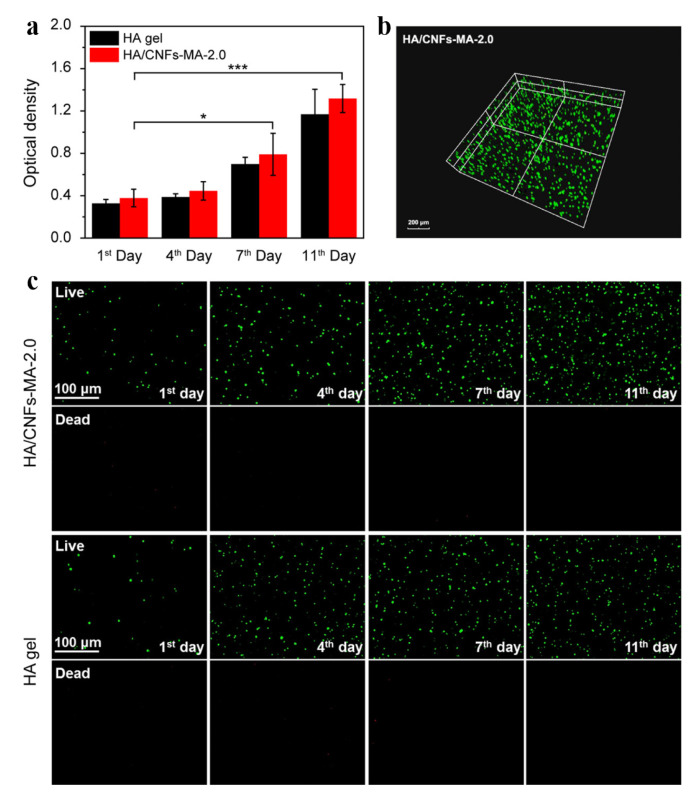
(**a**) The proliferation of BMSCs within the hydrogels at certain time points. (**b**) Three-dimensional distribution of BMSCs in the HA/CNFs-MA-2.0 nanocomposite hydrogel on the seventh day. (**c**) Live/dead staining of BMSCs in the HA hydrogel and the HA/CNFs-MA-2.0 nanocomposite hydrogel at certain time points, respectively. Data reproduced from ref. [140].

Articular cartilage consists of three regions: superficial, intermediate and deep, resulting in anisotropic mechanical properties of cartilage. Inspired by the structure of articular cartilage, Wang et al. [96] combined cellulose fabric, CNF and WC with biocompatible and biodegradable polyethylene glycol diacrylate (PEGDA) polymer matrices, respectively, to form superficial, intermediate and deep layers by UV polymerization. The composite hydrogel can adjust the morphology, orientation and phenotype of cultured cells, which indicates that the multi-zone composite hydrogel is a promising cartilage repair material. Current hydrogels do not have sufficient mechanical strength, fatigue strength and abrasion resistance under cyclic loading and wear conditions to be used as cartilage substitutes. Yang et al. [95] prepared composite hydrogels by infiltrating BC nanofiber networks with polyvinyl alcohol (PVA)-poly (2-acrylamido-2-methyl-1-sodium propanesulfonate) (PAMPS) double network hydrogels. BC provides tensile strength that is similar to collagen in cartilage, PAMPS provides sustained shear and compressive loading similar to aggrecan in cartilage and PVA provides elastic recovery and prevents stress concentration of individual BC fibers. The BC-PVA-PAMPS hydrogel has a proteoglycan modulus (0.78 Mpa) and permeability (3.2 × 10^−15^ m^4^ N^−1^ s^−1^), which gives it the same time-dependent mechanical response as cartilage under constrained compression. The friction coefficient (0.06) of BC-PAV-PAMPS hydrogel is about half that of cartilage, and its wear resistance is 4.4 times that of PVA. After 100,000 cycles, the BC-PAV-PAMPS hydrogel shows fatigue strength comparable to that of cartilage. These properties make the hydrogel an excellent repair material for cartilage damage. The scaffold made of pure BC has poor shape recovery performance, which limits its application in cartilage tissue engineering. Wang et al. [139] successfully prepared a green BC/SF double network hydrogel by soaking BC in a silk fibroin (SF) aqueous solution without using any crosslinking agent. The breaking strength and compressive strength of the hydrogel were both higher than that of pure BC hydrogel, and the hydrogel had good biocompatibility. Zhao et al. [140] prepared CNF/HA nano-composite hydrogel by photocrosslinking of methacrylate (MA)-functionalized CNF and methacrylate hydroxyapatite (HA). The compressive modulus of the composite hydrogel was 0.198.46 MPa ± 0.05 MPa, which showed sufficient compressive strength (0.198 MPa ± 0.009 MPa) and repairability. In addition, as shown in Figure 14, the proliferation of bone marrow mesenchymal stem cells in HA/CNFs-MA-2.0 hydrogels was better than that in HA hydrogels because CNF could promote cell adhesion, diffusion and growth.

## 5. Conclusions

As a sustainable resource, cellulose meets the requirements of biomedical materials, so the continuous development of functional cellulose-based materials is the future development direction. There is no doubt that cellulose materials have good application prospects in the field of tissue engineering due to their excellent physical and chemical properties, biocompatibility, biodegradability, low cytotoxicity and controllable reactivity. As a kind of tissue engineering scaffold material, the hydrogel is a useful and powerful tool to regulate the interaction between biomaterials and tissues/organs. In this paper, the structure and properties of cellulose and its derivatives from different sources were reviewed, and the preparation schemes of different cellulose-based hydrogels were studied. Because of the unique functions and characteristics of human tissues, the feasibility of cellulose-based hydrogels in tissue engineering was studied in this paper from the aspects of skin, bone and cartilage.

Although hydrogels based on cellulose and its derivatives have been extensively studied, there are still many challenges and difficulties to overcome in their biomedical applications. Firstly, the long-term biological safety of cellulose hydrogels prepared in biomedicine has not been systematically evaluated. Although natural cellulose is biocompatible and non-toxic, most studies have been based on cellular experiments or short-term histopathological studies. Therefore, the long-term toxicity and biocompatibility of cellulose-based hydrogels in tissue engineering should be confirmed in clinical animals. Secondly, the healing/repair process of various tissues is complex and multi-stage overlapping, while the common cellulose-based hydrogel can only play a role in a certain stage, so it is necessary to explore multifunctional hydrogel scaffold materials to expand their application range. In addition, the development of modern intelligent hydrogels with environmental responsiveness (pH, temperature, or humidity), and the realization of drug-release or synergistic effects to improve their performance under environmental stimulation have a good clinical application prospects. Thirdly, cellulose-based hydrogels lack innate antibacterial, antioxidant and regenerative activities, and thus cellulose-based composites need to be impregnated or doped with antibiotics or additives to enhance their biological activity. Fourthly, the large-scale production and clinical application of cellulosic hydrogels remain a problem, as they require a large number of experiments to verify, enabling the development of many more promising biomedical products. In summary, cellulosic materials have shown promising applications in recent tissue engineering fields, and we expect to provide the impetus for the development of functionalized cellulose-based hydrogels in various biomedical applications.

## Figures and Tables

**Table 1:** The differences between plant cellulose and bacterial cellulose.

Properties	Plant Cellulose	Bacterial Cellulose
purity	moderate to low	about 100%
crystallinity degree	54–88%	65–79%
water retention	60%	98%
degree of polymerization	ranges from 500–15,000	800–10,000

**Table 2:** Comparison of the mechanical properties of native tissues (skin, bone and cartilage) and cellulose-based engineered tissues.

Native Tissues	Cellulose-Based Engineered Tissues	Crosslinking Methods	Mechanical Properties	Reference
skin	Bisaldehyde-modified CNC and carboxymethyl chitosan (CMC) were crosslinked by Schiff base	Chemical crosslinking	The maximum storage modulus (G′) for hydrogel was around 4 kPa.	[91]
skin	Sodium alginate (SA)/NaCMC blend hydrogel	Physical crosslinking	Hydrogels’ tensile break stresses were more than 200 KPa, which is close to the tensile break stresses of mouse skin (240 KPa), rabbit skin (265 KPa) and human skin (150 KPa).	[92]
bone	CA nanofibers were deacetylated and mixed with chitosan (CS) to prepare composite hydrogels	Chemical crosslinking	The maximum compressive strength of the composite hydrogel was 30.19 kPa.	[93]
bone	Calcium phosphate (CaP)- incorporated bacterial cellulose (BC)-polyvinylpyrrolidone (PVP) based hydrogel scaffolds	Physical crosslinking	The compressive strengths of CaP/BC-PVP hydrogel scaffolds were found between 0.21 and 0.31 MPa, which is comparable with the human trabecular bone.	[94]
cartilage	Poly (vinyl alcohol)(PVA)-poly (2-acrylamido-2-methyl-1-sodium propanesulfonate) Polyvinyl alcohol (PVA)—poly (2-acrylamido-2-methyl-1-sodium propanesulfonic acid) (PAMPS) double network hydrogel permeated BC nanofiber network to prepare composite hydrogel	combination of physical and chemical crosslinking	The modulus values of the BC-PVA-PAMPS hydrogel (approximately 0.78 MPa) were in the range of values of human cartilage (0.46–1.43 MPa), the permeability of the hydrogel (3.2 × 10^−15^ m^4^ N^−1^ s^−1^) was also in the range of human cartilage permeability (1.2–9.2 × 10^−15^ m^4^ N^−1^ s^−1^), and the hydrogel deformed with time under lateral compression in the same manner as cartilage.	[95]
cartilage	Cellulose fabric, CNF and WC were combined with polyethylene glycol diacrylate (PEGDA) matrix, respectively, to form a three-layer hydrogel with a superficial layer, a middle layer and a deep layer	combination of physical and chemical crosslinking	The composite hydrogel formed naturally articular cartilage with region-dependent, nonlinear and viscoelastic properties. The compression modulus of the shallow, middle and deep layers were 298 kPa, 182 kPa and 9.8 Mpa, respectively.	[96]

## Data Availability

No new data were created or analyzed in this study. Data sharing is not applicable to this article.
